# Mapping the relationship between perceived involution and subjective well-being in Chinese university students: a network analysis across gender and grade levels

**DOI:** 10.3389/fpsyg.2025.1628064

**Published:** 2025-12-04

**Authors:** Chen Li

**Affiliations:** School of Psychology, Shandong Normal University, Jinan, China

**Keywords:** perceived involution, subjective well-being, network analysis, gender and grade differences, university students

## Abstract

Involution, as a rising sociocultural phenomenon in contemporary Chinese society, has raised concerns about its potential psychological impact on young people. However, little is known about how perceived involution relates to university students’ well-being and whether such patterns differ by gender and academic stage. The present study investigated perceived involution and subjective well-being among 5,235 university students using network analysis. Distinct characteristics of involution perception were identified across gender and academic year. Network structures revealed largely negative associations between perceived involution and well-being, with female students displaying denser and more interconnected networks than males. Additionally, comparisons across grade levels indicated differentiated psychological profiles: lower-grade students were more affected by academic stress, while upper-grade students showed stronger links between competitive norms and future-related concerns. These findings contribute to a nuanced understanding of how involution manifests psychologically and underscore the value of network analysis in mapping complex relationships between cultural stressors and mental health in youth populations.

## 1 Introduction

In recent years, “involution” has transformed from an obscure sociological term to a phenomenal buzzword (Li, 2025; Liu, 2021). It was developed to depict an anthropological framework for characterizing a society which cannot evolve nor stabilize itself but can only complicate its internal elements (Goldenweiser, 1937). Involution (also known as Chinese word, “Neijuan”) has disseminated to nearly all walks of life in mainland China in the recent few years, especially in terms of higher education (Cai, 2022; Li, 2021). Involution among college students refers to their unnecessary over-engagement or irrational competition to achieve academic performance or gain an edge in the job market (Kang and Jin, 2020; Zhou, 2024). It reflects a life of being overworked, stressed, anxious and feeling trapped, a lifestyle where many college students face negative consequences in this highly competitive environment yet receive little to no reward, even at the expense of other aspects of life (Wang et al., 2024; Yang et al., 2023). Perceived involution represents an individual’s subjective experience and cognitive appraisal of such atmosphere (Yu et al., 2022). While high levels of perceived involution may adversely affect college students’ mental health and well-being, few studies have examined the underlying mechanisms.

Subjective well-being refers to “a person’s cognitive and affective evaluations of his or her life” (Diener, 1984; Diener et al., 2002), it is an vital factor to mental health beyond merely the absence of mental illness and disorders. Subjective well-being is conceptualized as a tripartite construct, evaluated through indicators of life satisfaction and positive affect at high levels, alongside negative affect at low levels (Busseri and Sadava, 2010; Diener, 1994). Empirical studies demonstrate that subjective well-being contributes to improved physical health, extended lifespan, stronger interpersonal connections, enhanced job productivity, and greater psychological resilience (De Neve et al., 2013). Individuals’ subjective well-being complexly associated with multiple factors range from genetics to societal conditions (Diener et al., 2018a). As a specific population navigating a critical stage of self-development and socialization, the mental health and subjective well-being of college students warrant attention (Cha, 2003; Huang and Zhang, 2022; Renshaw and Bolognino, 2016). Especially in the context of increasing involution in higher education, exploring the impact of college students’ perception of involution on their subjective well-being contributes to enhancing their overall well-being.

Theoretical research has highlighted the negative effects of involution on the individual well-being among college students (Lu et al., 2024); however, empirical evidence remains limited. Previous studies have shown that being in an atmosphere of academic involution increases college students’ experience of relative deprivation (Liu et al., 2024a), making them more prone to dissatisfaction, stress, and frustration in the context of social comparison (Crosby, 1976; Smith et al., 2011). Consequently, the relative deprivation compels students to passively engage in more involution behaviors which lead to severe stress response (Liu et al., 2024a). Unlike achievement-motivated involution which negatively predict anxiety, passive involution (also referred to as hostage involution) is positively associated with anxiety among college students (Yi et al., 2022). Additionally, involution adversely affects students’ sense of self-worth and the quality of peer relationships, placing a burden on their psychological and social well-being (Liu, 2024). Moreover, academic involution exacerbates mental internal friction by intensifying academic stress, especially among students with high levels of rumination (Liu et al., 2024b). Form a perspective of relative deprivation theory, individuals feel deprived and dissatisfied not from an absolute lack of resources, but from perceiving themselves as disadvantaged compared to a relevant reference group (Smith et al., 2011). The competitive atmosphere of involution fosters upward social comparisons, and when students perceive inequity between their efforts and outcomes relative to peers, the resulting sense of relative deprivation directly leads to severe stress response and undermines subjective well-being (Liu et al., 2024a). However, researches traditionally treat these elements as distinct variables in a linear causal chain. We argue that involution may not act as a single uniform construct, but rather as a constellation of interrelated experiences that jointly undermine well-being according to network theory, which provides a stronger framework by conceptualizing psychological constructs as dynamic systems of mutually reinforcing components (Robinaugh et al., 2020). Applying this perspective allows us to move beyond examining isolated associations and instead reveal how involution-related factors interact to shape students’ subjective well-being.

Moreover, the impact of involution on subjective well-being among college students may vary across different genders and academic years. From a gender perspective, significant differences exist between males and females in terms of structural, sociocultural, and physiological factors that influence subjective well-being (Diener et al., 2018b). Additionally, the effects of these factors on subjective well-being differ by gender. For instance, some studies suggest that social pressure, particularly financial stress, has a stronger impact on men’s subjective well-being than on women’s (Alarcón-García et al., 2022), whereas access to infrastructure has been found to be more critical for women’s subjective well-being (Alexander et al., 2021). From an academic year perspective, lower-year and upper-year college students experience distinct stressors and involution contexts (Saleh et al., 2017). Lower-year students tend to focus more on their academic performance (Baghurst and Kelley, 2014), while upper-year students are more anxious about their future development and post-graduation prospects (Gao et al., 2020). Taken together, incorporating both gender and academic year factors is essential when examining the impact of involution on subjective well-being.

In summary, college students’ perception of involution may influence their well-being, with potential variations across gender and academic year. Statistically, moderation or mediation models are typically regarded as latent variable modeling, focusing solely on examining the relationships between observed variables and latent constructs without making inferences about interactions between different factors. Researchers introduced a novel network approach for studying psychological factors (Borsboom and Cramer, 2013). This data-driven method conceptualizes factors as dynamic systems rather than focusing on constructing latent variables (David et al., 2018). Network comparison analysis further provides a unique advantage by allowing direct statistical tests of differences in network structures across groups. Specifically, it enables the examination of whether the overall connectivity strength, global structure, or individual edge weights differ between populations, offering a rigorous approach to identifying group-specific patterns (Robinaugh et al., 2020). Consequently, this approach has been increasingly applied to research on college students’ mental health and well-being (Govorova et al., 2020; Ma et al., 2022; Zhou et al., 2022). Based on network analysis, the present study aims to investigate the impact of college students’ perception of involution on their subjective well-being, as well as the roles of gender and academic year.

## 2 Methods

### 2.1 Participants

A total of 5,235 college students provided valid questionnaires and were included in the present study (mean age = 20.00 ± 1.29, 3,715 females; mean age of females = 20.00 ± 1.26, mean age of males = 19.93 ± 1.35, Table 1). Among them, 3,230 were first-year students (61.7%, 2,205 females), 1,439 were third-year students (27.5%, 1,117 females), and 566 were fourth-year students (10.8%, 393 females). Second-year students were not included because of recruitment constraints at the time of data collection. In this study, third- and fourth-year students were categorized as upper-year students (*n* = 2,005, 38.3%). The sample was drawn from multiple universities across various regions and majors in China. Participants were recruited via internet and completed the questionnaire online. Those who completed the questionnaire with acceptable data (quality verified by two attention-check items) received a monetary reward. Data collection took place between November 2024 and February 2025. Electronic informed consent was obtained from all participants. This study received approval (SDNU2024087) from the local ethics committee of Shandong Normal University.

**Table 1:** Sample characteristics in the present study.

	Whole-sample	Gender				Academic year			
		Female	Male	*t*	*p*	First-year	3rd- and 4th-year	*t*	*p*
*n*	5,235	3,715	1,520			3,230	2005		
Age	20.00 ± 1.29	20.00 ± 1.26	19.93 ± 1.35	1.65	0.099	19.11 ± 0.54	21.38 ± 0.82		
IPN	4.04 ± 0.74	4.06 ± 0.72	4.01 ± 0.8	2.38	0.018	4.04 ± 0.76	4.06 ± 0.71	−0.98	0.329
IPN_PS	2.81 ± 1.17	2.84 ± 1.15	2.75 ± 1.21	2.45	0.015	2.92 ± 1.19	2.65 ± 1.11	8.14	< 0.001
IPN_SN	4.75 ± 1.21	4.79 ± 1.17	4.67 ± 1.31	3.17	0.002	4.68 ± 1.23	4.88 ± 1.18	−5.7	< 0.001
IPN_CB	5.13 ± 1.02	5.13 ± 0.97	5.12 ± 1.13	0.46	0.647	5.06 ± 1.04	5.23 ± 0.97	−5.67	< 0.001
IPN_RS	3.52 ± 1.31	3.52 ± 1.26	3.52 ± 1.42	0.04	0.969	3.51 ± 1.33	3.53 ± 1.28	−0.59	0.556
IWB	11.49 ± 2.39	11.49 ± 2.32	11.49 ± 2.56	0.05	0.963	11.53 ± 2.44	11.42 ± 2.31	1.55	0.121

IPN, individuals’ perceptions of involution; IPN_PS, psychological stress; IPN_SN, social norms; IPN_CB, competitive behavior; IPN_RS, resource scarcity; IWB, subjective well-being.

### 2.2 Measurements

This study employed the Individuals’ Perceptions of Involution (IPN) Scale, which captures subjective experiences of Neijuan, to evaluate college students’ perceptions of this phenomenon (Zhang, et al. 2024). The scale consists of 18 items and encompasses four dimensions (Table 2): psychological stress (items 1–5, e.g., “I feel frustrated in my study/work”), social norms (items 6–9, e.g., “Most people around me continue to take on additional work even after completing the minimum requirements”), competitive behavior (items 10–14, e.g., “People around me gain recognition from others through competition”), and resource scarcity (items 15–18, e.g., “Limited resources in my environment negatively affect my interpersonal relationships”). Responses were rated on a 7-point Likert scale. Higher scores on both the subscales and the total scale indicate a stronger perception of involution. In previous studies, the internal consistency coefficients (Cronbach’s α) for all dimensions exceeded 0.70 (Yang et al., 2023). In the present study, the scale demonstrated good internal consistency, with Cronbach’s α coefficients of 0.85 for the total scale and 0.86, 0.87, 0.92, and 0.89 for its four subscales, respectively. Confirmatory factor analysis supported the four-factor structure of the IPN scale, showing good fit, *χ*^2^(129) = 8026.76, *p* < 0.001, CFI (comparative fit index) = 0.88, TLI (Tucker–Lewis index) = 0.86, RMSEA (root mean square error of approximation) = 0.11, SRMR (standardized root mean square residual) = 0.07.

**Table 2:** The specific items of the IPN and IWB scales.

Individuals’ perceptions of involution	
IPN1	I feel full of energy when I am studying/working.
IPN2	In my study/work, I think I’m doing something I truly love.
IPN3	Studying/working makes me feel down.
IPN4	I feel frustrated during my study/work.
IPN5	In my study/work, I feel nervous or “on the verge of being overwhelmed.”
IPN6	Most people around me will continue to do more work even after they have completed the minimum task requirements.
IPN7	Most people around me have gotten used to exceeding the work/study task requirements.
IPN8	Just meeting the minimum standard of tasks in study and work is not enough; most people will keep striving to do more.
IPN9	Most people around me show their attitude toward work/study by making more and excessive efforts than others.
IPN10	The people around me become outstanding through competition.
IPN11	The people around me can gain a good social status through competition.
IPN12	The people around me have gained the recognition of others through competition.
IPN13	The people around me have received comprehensive training through competition.
IPN14	The people around me will all strive to win every competition.
IPN15	The limited resources in my environment have had an adverse impact on my interpersonal relationships.
IPN16	There are too few resources in my environment, so I cannot get the rewards I deserve.
IPN17	Due to the insufficient available resources in the environment, I cannot handle important matters properly.
IPN18	Compared with the efforts and sacrifices I have made, my life should be better than it is now.

Subjective well-being	
IWB1	Bored vs. Interesting
IWB2	Happy vs. Painful
IWB3	Useless vs. Valuable
IWB4	Having Many Friends vs. Lonely
IWB5	Fulfilled vs. Empty
IWB6	Hopeless vs. Hopeful
IWB7	Depressed vs. Rewarded
IWB8	Life Treats Me Very Well vs. Life Does not Give Me Any Opportunities
IWB9	Totally Satisfied with Life in General vs. Totally Dissatisfied with Life in General

All reverse-scored items have been recoded, with higher IPN scores indicating greater perceived involution and higher IWB scores reflecting greater subjective well-being. IPN, individuals’ perceptions of involution; IWB, subjective well-being.

Subjective well-being among college students was measured using the index of well-being (IWB) developed by Zhang et al. (2024). The scale consists of two components (Table 2). The first part assesses the affective dimension of subjective well-being through eight items, each rated on a 7-point semantic differential scale anchored by opposing adjectives (e.g., “happy–unhappy,” “pleasant–unpleasant”). These items capture the respondent’s positive and negative emotional experiences. The ninth item evaluates the cognitive dimension of subjective well-being by assessing overall life satisfaction (e.g., “Which number best represents your overall satisfaction with life?”), also rated on a 7-point scale. An individual’s overall subjective well-being score is calculated by applying different weights to the affective and cognitive components. In the current study, the overall well-being scale showed high reliability, with a Cronbach’s *α* of 0.94. Confirmatory factor analysis further supported the two-factor structure of the IWB and showed good fit, *χ*^2^(27) = 1142.17, *p* < 0.001, CFI = 0.97, TLI = 0.96, RMSEA = 0.09, SRMR = 0.024.

### 2.3 Network analyses

After necessary data cleaning, missing value imputation, outlier processing, and descriptive analyses, network analysis was conducted using R in the present study. The network structure was estimated using the graphical LASSO method with Extended Bayesian Information Criterion (EBIC) model selection, implemented via the estimateNetwork function. The network consisted of 27 nodes, representing perceived involution (18 nodes) and subjective well-being items (9 nodes). In the present study, the network was constructed at the item level rather than the dimension level. Item-level networks allow for a more fine-grained analysis of the interactions between specific involution-related perceptions and well-being indicators, which may otherwise be obscured when aggregating items into dimensions.

Connections between nodes (edges) were visualized using the qgraph package, with edge thickness indicating the strength of the association and color denoting the direction (blue = positive association, red = negative association). Centrality metrics were computed to assess the relative importance of each node. Strength centrality was calculated as the sum of the absolute weights of all edges connected to a node, reflecting its overall influence in the network (McNally, 2016). Betweenness centrality measured how often a node lay on the shortest path between pairs of other nodes (Bringmann et al., 2019), whereas closeness centrality captured the inverse of the average shortest path length from a node to all others.

Bridge nodes connecting the two communities (perceived involution and subjective well-being) were identified using the bridge function from the networktools package. Bridge strength was computed as the sum of the absolute weights of edges linking a node to nodes in the other community (Brown et al., 2020).

Network stability was evaluated using the bootnet package. Edge-weight accuracy was assessed through nonparametric bootstrapping to generate 95% confidence intervals. Centrality stability was evaluated via correlation stability (CS) coefficients, with a CS coefficient of ≥0.25 considered the minimum threshold for interpreting centrality estimates with adequate reliability (Epskamp et al., 2018).

To examine potential differences in network structure across subgroups, we conducted Network Comparison Tests (NCT). Separate analyses compared networks between genders (male vs. female) and across grade levels (fresh vs. uppers). The NCT assessed both global strength invariance and specific edge-weight differences, with statistical significance determined through permutation testing (1,000 iterations). This approach allowed us to explore whether the network structure varied systematically by demographic factors.

## 3 Results

### 3.1 Differences in IPN and IWB scores across gender and grade level

The descriptive statistics of IPN and IWB scores by gender and grade level are presented in Table 1. Results indicated that females reported significantly higher levels of perceived involution than males. Specifically, females reported greater perceived psychological pressure and higher experiences of social norms and comparisons. No significant differences in overall perceived involution were found between lower- and upper-grade university students. However, this pattern was driven by opposite trends in two subdimensions: lower-grade students experienced higher psychological pressure, whereas upper-grade students reported greater experiences of social norms and competitive behaviors. No significant differences in subjective well-being were observed across gender or grade level.

### 3.2 Overall network characteristics

**Figure 1:**
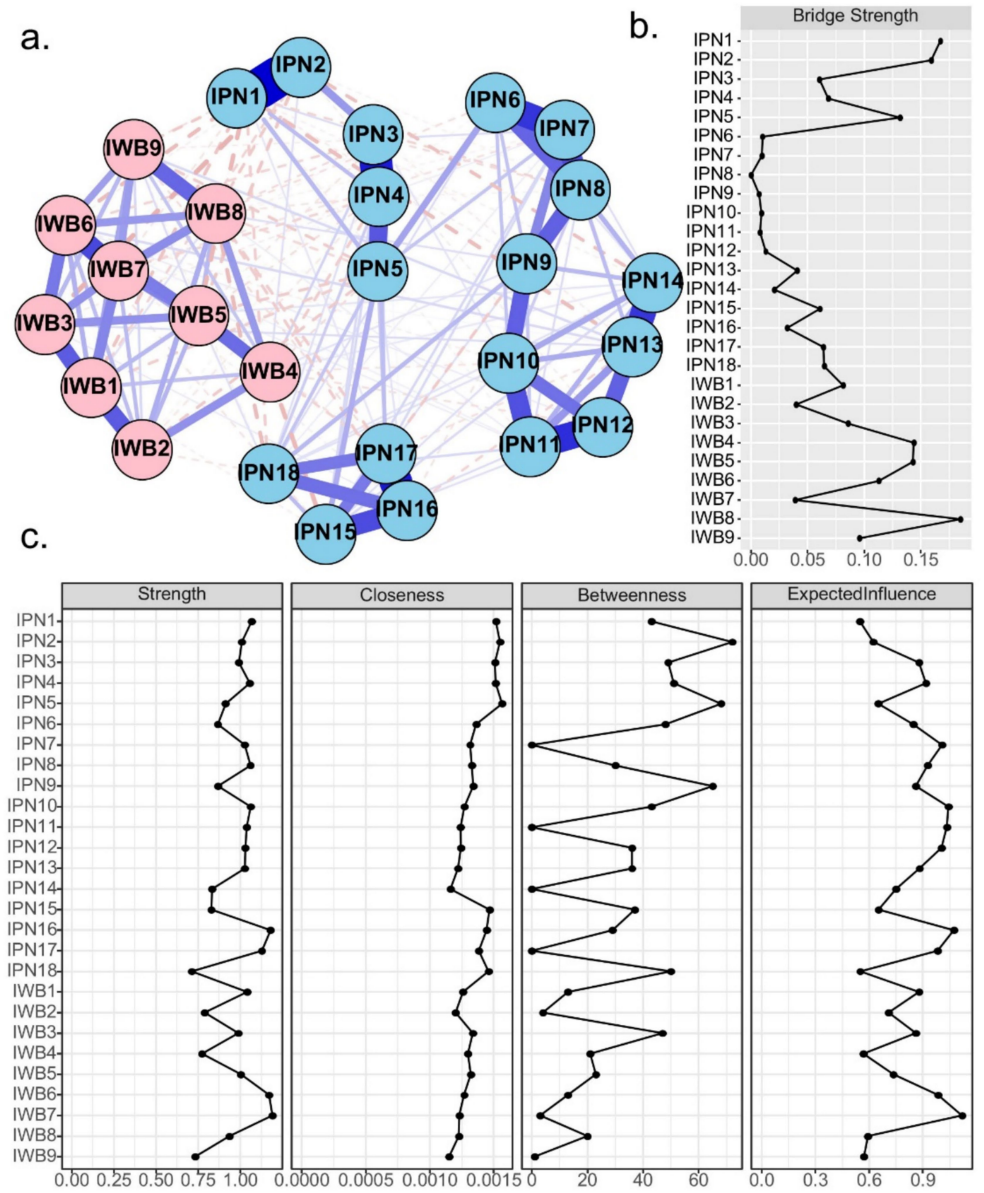
**(a)** Network structure of perceived involution and subjective well-being, IPN represents the perceptions of involution questionnaire, IWB represents the subjective well-being. Edge thickness indicating the strength of the association and color denoting the direction (i.e., blue = positive association, red = negative association). **(b)** Standardized bridge strength of each node. **(c)** Centrality measures of all nodes within the network.

Figure 1 presents the estimated network structure of perceived involution (IPN) and subjective well-being (IWB) across all participants. Supplementary Table S3 shows the weighted edges among the 27 nodes. Of the 351 possible edges, 189 (53.8%) were nonzero. Among the 162 potential cross-community edges (between IPN and IWB), 60 edges (37.0%) were nonzero. Among these 60 edges, 48 (80.0%) were negative, Indicating an overall negative association between perceived involution and subjective well-being.

Centrality indices including strength, betweenness, and closeness are displayed in Figure 1. In terms of strength, IPN item 16 and IWB items 6 and 7 showed the highest centrality. Regarding closeness, IPN items 1, 2, 3, 4, and 5 exhibited the highest values. For betweenness, IPN items 2 and 5 showed the highest values, suggesting that these nodes may act as critical connectors or “hubs” within the network. In terms of bridge strength, IPN items 1 and 2, along with IWB item 8, demonstrated the highest bridge strength, indicating their key roles in linking perceived involution and subjective well-being communities.

### 3.3 Gender differences in network characteristics

**Figure 2:**
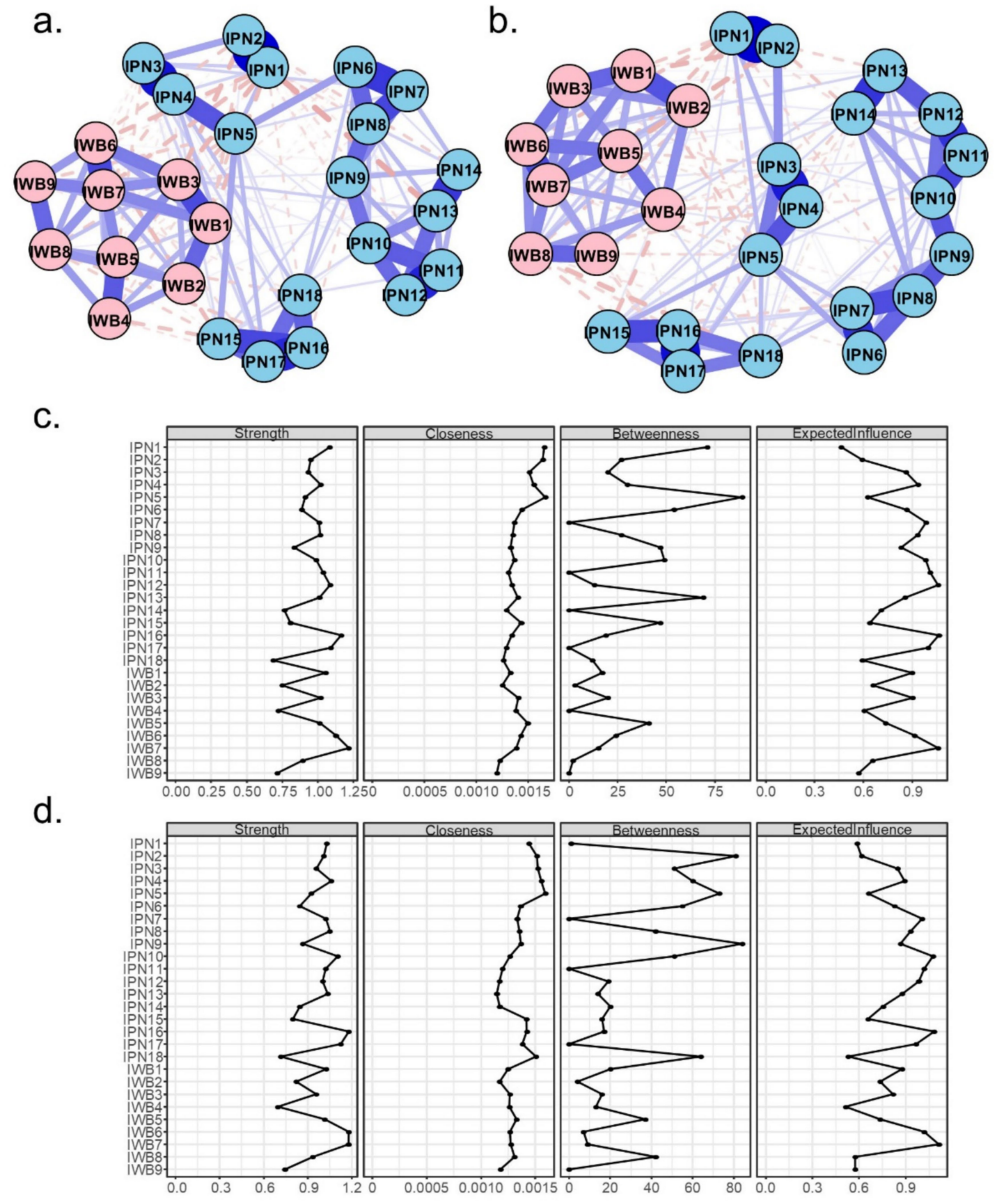
Network structure of perceived involution and subjective well-being on male **(a)** and female **(b)**. Panels **(c,d)**, respectively, display the node centrality indices of the male and female networks.

Figure 2 presents the estimated network structures and centrality indices separately for male and female participants. Supplementary Table S1 summarizes the edge weights for each group.

Among females, 176 (50.1%) of the 351 possible edges were nonzero. Of the 162 cross-community edges, 55 (34.0%) were nonzero, with 44 edges (80.0%) being negative. Among males, 154 (43.9%) of the 351 edges were nonzero. Of the 162 cross-community edges, 43 (26.5%) were nonzero, with 36 edges (83.7%) being negative.

**Figure 3:**
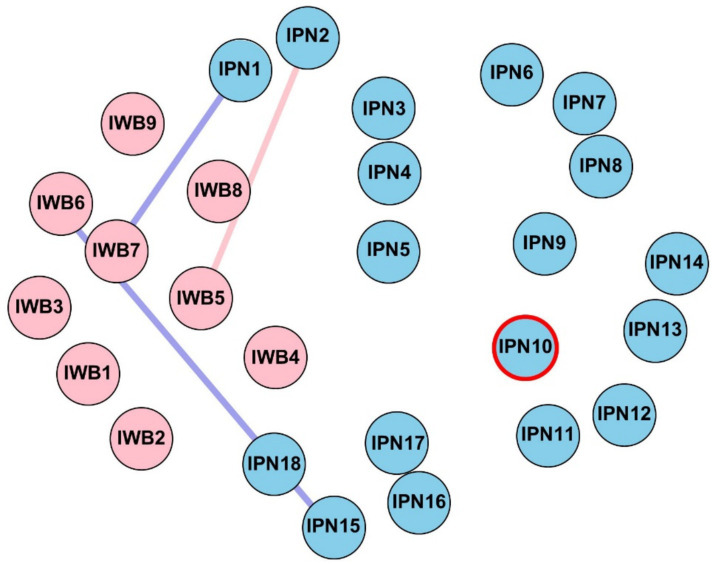
The comparison of edge weights and bridge strength centrality between the male and female networks. Only edges connecting the two communities (IPN and IWB) are shown if they significantly differ between networks. Blue edges indicate stronger connections in the male network, while red edges represent stronger connections in the female network. Nodes highlighted with red borders indicate significantly higher bridge strength in the female network.

Edge-weight comparisons revealed several significant gender differences, as shown in Figure 3. Specifically, significant cross-community differences were observed for edges between IPN2 and IWB5, IPN15 and IWB6, and IPN1 and IWB7.

Node-level analyses showed that the strongest node in terms of strength for males was IWB item 7 and IPN item 16. For females, the strongest nodes were IWB items 6 and 7, as well as IPN item 16. Regarding closeness, the highest values for males were found in IPN items 1, 2, and 5, whereas for females, IPN items 3, 4, and 5 were the highest. In terms of betweenness, IPN item 5 was the highest for males, while IPN items 9 were the highest for females.

Comparisons of node centrality indices indicated that males and females differed significantly in strength at IPN10 and in betweenness at IPN13. Specifically, females exhibited higher strength at IPN10 (competition to become outstanding), whereas males showed higher betweenness at IPN13 (competitions leading to comprehensive development). No significant differences were found in bridge strength between genders.

### 3.4 Grade-level differences in network characteristics

**Figure 4:**
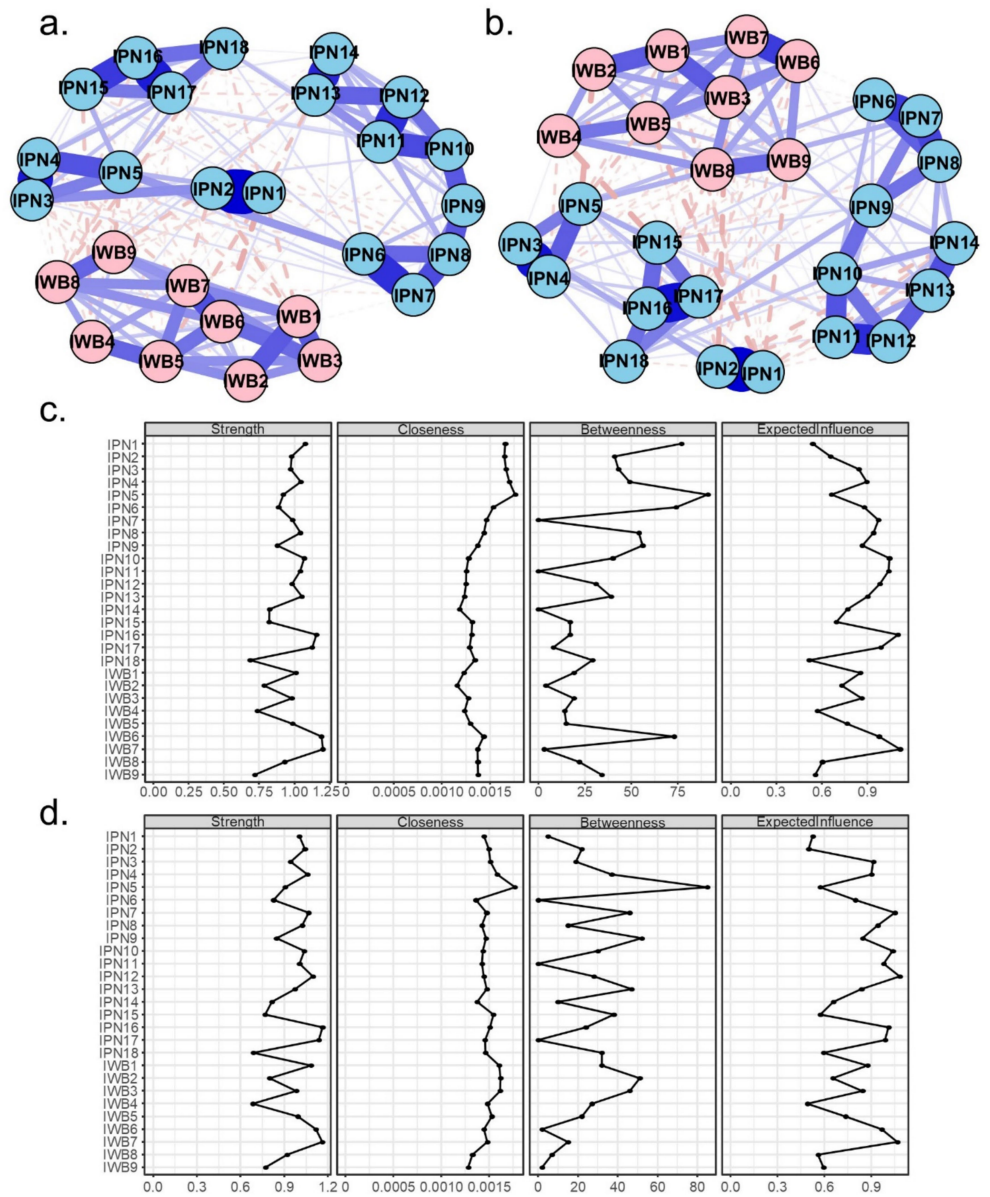
Network structure of perceived involution and subjective well-being on lower- **(a)** and upper-grade **(b)** students. Panels **(c,d)**, respectively, display the node centrality indices of the lower- **(a)** and upper-grade **(b)** networks.

**Figure 5:**
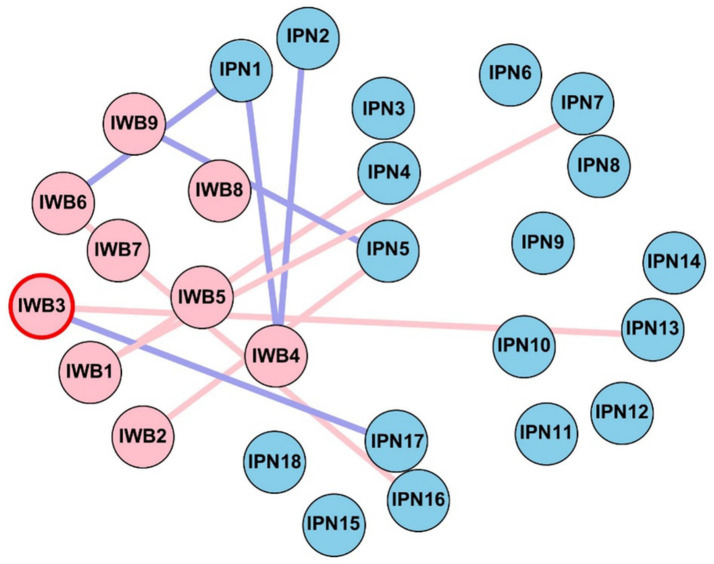
The comparison of edge weights and bridge strength centrality between the lower-grade and upper-grade networks. Only edges connecting the two communities (IPN and IWB) are shown if they significantly differ between networks. Blue edges indicate stronger connections in the lower-grade network, while red edges represent stronger connections in the upper-grade network. Nodes highlighted with red borders indicate significant higher bridge strength in the upper-grade network.

Figure 4 presents the estimated network structures and centrality indices separately for lower-grade and upper-grade students. Supplementary Table S2 summarizes the edge weights for each group. Among lower-grade students, 170 of the 351 possible edges were nonzero (48.4%). Of the 162 cross-community edges, 54 were nonzero (33.3%), with 45 edges being negative (83.3%). Among upper-grade students, 173 of the 351 edges were nonzero (49.3%). Of the 162 cross-community edges, 57 were nonzero (35.2%), with 45 edges being negative (79.0%). Edge-weight comparisons revealed 27 significant differences across grade levels, 10 of which were cross-community edges (Figure 5).

Node-level analyses showed that the nodes with the highest strength among lower-grade students were IWB items 6 and 7, while among upper-grade students, the highest strength nodes were IWB item 7 and IPN item 16. Regarding closeness, IPN items 4 and 5 had the highest values among lower-grade students, while IPN item 5 had the highest closeness among upper-grade students. For betweenness, IPN item 5 was the highest in both lower- and upper-grade networks. Comparisons of node centrality indices indicated that no significant differences were observed in strength between grade levels. Lower- and upper-grade students differed significantly in bridge strength at IWB3.

## 4 Discussion

The present study investigated the perceived involution and subjective well-being among university students across gender and grade levels using network analysis. First, the study identified distinct characteristics of involution perception across gender and academic year. Network analysis revealed that perceived involution and subjective well-being were largely negatively associated, with denser and more interconnected networks observed among females compared to males. Furthermore, notable differences emerged in network structures across grade levels, highlighting distinct patterns of psychological experience in different academic stages.

### 4.1 Gender-specific links between involution and well-being

A closer examination of gender differences showed that in the female sample, more edges existed between perceived involution and subjective well-being nodes, and a larger proportion of these connections were negative. Prior research has consistently found that women report higher emotional reactivity and are more likely to engage in emotion-focused coping when facing stressors (Domes et al., 2010; Givon et al., 2023), which may make them more vulnerable to negative psychological outcomes when exposed to competitive or resource-scarce environments (Schilling et al., 2022; Stumps et al., 2024). Specifically, items reflecting environmental resource scarcity and emotional distress exhibited the highest strength centrality among females, underscoring the central role of emotional and resource-related appraisals in linking involution and well-being. These findings are consistent with evidence that women tend to evaluate their environments in more affectively laden ways and are more likely to experience hopelessness in the face of goal obstruction or resource deprivation (Givon et al., 2023; Thomson et al., 2021).

Practically, interventions targeting female students could focus on alleviating emotional distress and resource-related concerns. For example, universities could provide emotion regulation training, counseling services, and guidance on realistic goal-setting to reduce the negative impact of involution. In contrast, male students might benefit from interventions emphasizing goal-setting, problem-solving strategies, and coping skills to manage academic and career pressures.

### 4.2 Developmental shifts in involution–well-being networks across grade levels

Network comparisons across grade levels highlight a developmental shift in the nature of perceived involution and its psychological impact. Although overall perceived involution levels did not differ significantly between lower- and upper-grade students, distinct patterns emerged within specific involution subdimensions and network structures. Lower-grade students displayed higher centrality in items related to psychological stress, suggesting that their involution experiences are predominantly centered on immediate academic performance and adaptation to the university life (Garett et al., 2017; Lee et al., 2009). This pattern reflects the fact that first-year students often face academic pressure from exams, coursework, and the struggle to establish effective learning strategies (Credé and Niehorster, 2012; Turner et al., 2023). In comparison, upper-grade students exhibited greater centrality in items associated with social norms and competitive behaviors, indicating a shift toward concerns about social evaluation, peer comparison, and external markers of achievement (Zheng et al., 2022). This may be due to increasing awareness of post-graduate transitions, such as employment, graduate school, and long-term career planning, which are highly salient among third- and fourth-year students (Chowdhury et al., 2022; Ma and Bennett, 2021). The bridge connections in the upper-grade network also showed stronger links between perceptions of limited resources and diminished subjective well-being, highlighting how concerns about future scarcity become more psychologically impactful as students near graduation (Ma et al., 2022; Zhou et al., 2022).

In practice, this indicates that interventions should be tailored by grade: lower-grade students may benefit most from academic adaptation support and learning skills training, whereas upper-grade students may require guidance on career planning, social comparison management, and resilience-building. Providing resources and counseling focused on future uncertainties could help mitigate anxiety and enhance well-being among senior students.

### 4.3 Strengths and limitations

This study has several strengths. First, it applied network analysis to examine the complex relationships between perceived involution and subjective well-being, identifying key nodes and bridge connections that reveal underlying psychological mechanisms. Second, it explored gender- and grade-specific patterns, providing insights into developmental and demographic variations in involution experiences. These findings have theoretical value by clarifying how involution affects well-being and highlighting the mechanisms linking these constructs. Practically, these results inform targeted interventions: for example, first-year students may benefit from academic adaptation support. By pinpointing the connections most strongly linked to reduced well-being, universities and counselors can more effectively allocate resources to alleviate emotional distress and perceived resource scarcity.

Despite the strengths of this study, several limitations should be acknowledged. First, the cross-sectional design precludes causal inferences about the relationships between perceived involution and subjective well-being. Longitudinal research is needed to examine how these networks evolve over time and whether certain nodes predict future psychological outcomes. Second, although network analysis offers a nuanced view of variable interrelations, the interpretation of node and bridge centralities should be approached with caution, as measurement noise and sample-specific characteristics may influence network structures. Third, the CFA results for the IPN scale were somewhat suboptimal, which may affect the robustness of the conclusions. Future studies should prioritize revising and improving the scale to enhance its validity and reliability. Forth, while involution has recently gained prominence as a sociocultural phenomenon in China, further cross-cultural research is needed to clarify its broader psychological and societal implications. Fifth, this study did not include second-year students due to recruitment constraints at the time of data collection, which may limit the generalizability of the findings across all grade levels.

## 5 Conclusion

In conclusion, this study advances our understanding of how perceived involution and subjective well-being are intertwined among university students, revealing important differences across gender and academic stage. These findings underscore the need for tailored interventions: addressing emotional coping and resource perceptions among females, and targeting academic stress management in lower-grade students while supporting future planning and resilience-building in upper-grade students. Moreover, the network perspective highlights the importance of specific psychological experiences—such as feeling energized and hopeful—as potential leverage points for enhancing well-being amid involution pressures. Future research should continue to explore these dynamics longitudinally and across diverse cultural contexts to inform more effective psychological and educational support strategies.

## Data Availability

The raw data supporting the conclusions of this article will be made available by the authors, without undue reservation.
