# Correction: Mapping the relationship between perceived involution and subjective well-being in Chinese university students: a network analysis across gender and grade levels

**DOI:** 10.3389/fpsyg.2026.1869173

**Published:** 2026-05-12

**Authors:** Chen Li

**Affiliations:** School of Psychology, Shandong Normal University, Jinan, China

**Keywords:** perceived involution, subjective well-being, network analysis, gender and grade differences, university students

An incorrect **Funding** statement was provided. The correct **Funding** statement reads:

“The author(s) declare that financial support was received for this work and/or its publication. This research was supported by the 2025 Humanities and Social Sciences Projects of Shandong Federation of Social Sciences and the 2025 Research Project of the China Youth Research Association (2025B19).”

The original version of this article has been updated.

