# Supplementary material for: Mapping the relationship between perceived involution and subjective well-being in Chinese university students: a network analysis across gender and grade levels
Source: Front Psychol. 2025 Dec 4;16:1628064. doi: 10.3389/fpsyg.2025.1628064 (PMC12713321; doi:10.3389/fpsyg.2025.1628064)
Supplement: Supplementary file 1 [file Table_1.docx]

**Mapping the Relationship Between Perceived Involution and Subjective Well-Being in Chinese University Students: A Network Analysis Across Gender and Grade Levels**

Chen Li^1^

1. School of Psychology, Shandong Normal University, Jinan, 250358, China

Supplementary Table S1 – S3

Table S1. Weighted edges among the 27 nodes of the male (a) and female (b) sample.

| (a) | | | | | | | | | | | | | | | | | | | | | | | | | | | |
| --- | --- | --- | --- | --- | --- | --- | --- | --- | --- | --- | --- | --- | --- | --- | --- | --- | --- | --- | --- | --- | --- | --- | --- | --- | --- | --- | --- |
|  | IPN1 | IPN2 | IPN3 | IPN4 | IPN5 | IPN6 | IPN7 | IPN8 | IPN9 | IPN10 | IPN11 | IPN12 | IPN13 | IPN14 | IPN15 | IPN16 | IPN17 | IPN18 | IWB1 | IWB2 | IWB3 | IWB4 | IWB5 | IWB6 | IWB7 | IWB8 | IWB9 |
| IPN1 |  |  |  |  |  |  |  |  |  |  |  |  |  |  |  |  |  |  |  |  |  |  |  |  |  |  |  |
| IPN2 | 0.682 |  |  |  |  |  |  |  |  |  |  |  |  |  |  |  |  |  |  |  |  |  |  |  |  |  |  |
| IPN3 | 0.028 | 0.084 |  |  |  |  |  |  |  |  |  |  |  |  |  |  |  |  |  |  |  |  |  |  |  |  |  |
| IPN4 | 0.019 | 0 | 0.563 |  |  |  |  |  |  |  |  |  |  |  |  |  |  |  |  |  |  |  |  |  |  |  |  |
| IPN5 | 0.043 | 0.004 | 0.152 | 0.296 |  |  |  |  |  |  |  |  |  |  |  |  |  |  |  |  |  |  |  |  |  |  |  |
| IPN6 | 0 | 0 | 0 | 0 | 0.095 |  |  |  |  |  |  |  |  |  |  |  |  |  |  |  |  |  |  |  |  |  |  |
| IPN7 | 0 | 0 | 0.023 | 0 | 0.016 | 0.408 |  |  |  |  |  |  |  |  |  |  |  |  |  |  |  |  |  |  |  |  |  |
| IPN8 | -0.019 | -0.005 | 0 | 0 | 0 | 0.249 | 0.349 |  |  |  |  |  |  |  |  |  |  |  |  |  |  |  |  |  |  |  |  |
| IPN9 | -0.001 | 0 | 0 | 0 | 0 | 0.047 | 0.143 | 0.234 |  |  |  |  |  |  |  |  |  |  |  |  |  |  |  |  |  |  |  |
| IPN10 | 0 | -0.002 | 0 | 0 | 0.008 | 0.031 | 0 | 0.047 | 0.204 |  |  |  |  |  |  |  |  |  |  |  |  |  |  |  |  |  |  |
| IPN11 | 0 | -0.013 | 0 | 0 | 0.003 | 0 | 0 | 0.024 | 0.064 | 0.273 |  |  |  |  |  |  |  |  |  |  |  |  |  |  |  |  |  |
| IPN12 | 0 | -0.013 | 0 | 0 | 0 | 0.003 | 0 | 0.006 | 0.030 | 0.230 | 0.496 |  |  |  |  |  |  |  |  |  |  |  |  |  |  |  |  |
| IPN13 | -0.072 | -0.005 | 0 | 0 | 0 | 0 | 0 | 0.046 | 0 | 0.144 | 0.083 | 0.225 |  |  |  |  |  |  |  |  |  |  |  |  |  |  |  |
| IPN14 | -0.002 | -0.025 | 0 | 0 | 0 | 0.011 | 0.047 | 0.020 | 0.059 | 0.035 | 0.063 | 0.082 | 0.406 |  |  |  |  |  |  |  |  |  |  |  |  |  |  |
| IPN15 | 0 | 0 | 0.005 | 0.013 | 0.080 | 0 | 0 | 0 | 0.018 | 0.014 | 0 | 0 | 0 | 0 |  |  |  |  |  |  |  |  |  |  |  |  |  |
| IPN16 | 0 | 0 | 0.021 | 0.003 | 0.010 | 0 | 0 | -0.017 | 0 | 0 | 0 | 0 | 0 | 0 | 0.346 |  |  |  |  |  |  |  |  |  |  |  |  |
| IPN17 | 0 | 0 | 0.019 | 0.044 | 0.049 | 0 | 0 | 0 | 0 | 0 | 0 | 0 | 0 | 0 | 0.234 | 0.473 |  |  |  |  |  |  |  |  |  |  |  |
| IPN18 | -0.014 | 0 | 0 | 0.040 | 0.012 | 0.032 | 0.009 | 0 | 0.035 | 0 | 0.013 | 0 | 0 | 0.003 | 0.013 | 0.258 | 0.224 |  |  |  |  |  |  |  |  |  |  |
| IWB1 | -0.046 | -0.030 | 0 | 0 | 0 | 0 | 0 | 0 | 0 | 0 | 0 | 0 | 0 | 0 | 0 | 0 | 0 | 0 |  |  |  |  |  |  |  |  |  |
| IWB2 | 0 | 0 | 0 | -0.009 | -0.030 | -0.002 | -0.005 | 0 | 0 | 0 | 0 | 0 | 0 | 0 | 0 | 0 | 0 | 0 | 0.242 |  |  |  |  |  |  |  |  |
| IWB3 | -0.004 | -0.053 | -0.002 | 0 | 0 | 0 | 0 | 0 | 0 | 0 | 0 | 0 | 0.025 | 0.008 | 0 | 0 | 0 | 0 | 0.333 | 0.071 |  |  |  |  |  |  |  |
| IWB4 | 0 | 0 | 0 | 0 | -0.013 | 0 | 0 | 0 | 0 | 0 | 0 | 0 | 0 | 0.002 | -0.035 | 0 | -0.009 | 0 | 0.059 | 0.103 | 0 |  |  |  |  |  |  |
| IWB5 | -0.055 | 0 | -0.023 | -0.004 | -0.039 | -0.007 | -0.006 | 0 | 0 | 0 | 0 | 0 | 0 | 0 | -0.003 | 0 | 0 | 0 | 0.029 | 0.096 | 0.127 | 0.281 |  |  |  |  |  |
| IWB6 | -0.047 | 0 | -0.001 | 0 | 0 | 0 | 0 | 0 | 0 | 0 | 0.004 | 0 | 0 | 0.001 | -0.045 | 0 | -0.013 | 0 | 0.086 | 0.032 | 0.174 | 0.045 | 0.140 |  |  |  |  |
| IWB7 | -0.044 | 0 | -0.005 | -0.029 | 0 | 0 | 0 | 0 | 0 | 0 | 0 | 0 | 0 | 0 | 0 | 0 | 0 | 0 | 0.147 | 0.048 | 0.132 | 0.071 | 0.117 | 0.349 |  |  |  |
| IWB8 | 0 | -0.002 | -0.005 | 0 | -0.030 | 0 | 0 | 0 | 0 | 0 | 0 | 0 | 0.002 | 0 | 0 | -0.032 | -0.024 | -0.025 | 0 | 0.111 | 0.040 | 0.103 | 0.085 | 0.080 | 0.113 |  |  |
| IWB9 | -0.005 | -0.031 | 0 | 0 | -0.028 | 0 | 0 | 0 | 0 | 0 | 0 | 0 | 0.001 | 0 | 0 | 0 | 0 | -0.007 | 0.081 | 0 | 0.051 | 0 | 0 | 0.109 | 0.161 | 0.240 |  |
| (b) | | | | | | | | | | | | | | | | | | | | | | | | | | | |
| IPN1 |  |  |  |  |  |  |  |  |  |  |  |  |  |  |  |  |  |  |  |  |  |  |  |  |  |  |  |
| IPN2 | 0.686 |  |  |  |  |  |  |  |  |  |  |  |  |  |  |  |  |  |  |  |  |  |  |  |  |  |  |
| IPN3 | 0.006 | 0.118 |  |  |  |  |  |  |  |  |  |  |  |  |  |  |  |  |  |  |  |  |  |  |  |  |  |
| IPN4 | 0.056 | 0.008 | 0.536 |  |  |  |  |  |  |  |  |  |  |  |  |  |  |  |  |  |  |  |  |  |  |  |  |
| IPN5 | 0.044 | 0 | 0.183 | 0.302 |  |  |  |  |  |  |  |  |  |  |  |  |  |  |  |  |  |  |  |  |  |  |  |
| IPN6 | 0 | 0 | 0.001 | 0 | 0.078 |  |  |  |  |  |  |  |  |  |  |  |  |  |  |  |  |  |  |  |  |  |  |
| IPN7 | 0 | 0 | 0 | 0.002 | 0.047 | 0.434 |  |  |  |  |  |  |  |  |  |  |  |  |  |  |  |  |  |  |  |  |  |
| IPN8 | -0.019 | 0 | -0.008 | 0 | -0.004 | 0.246 | 0.327 |  |  |  |  |  |  |  |  |  |  |  |  |  |  |  |  |  |  |  |  |
| IPN9 | 0 | 0 | 0 | 0 | 0 | 0.034 | 0.154 | 0.276 |  |  |  |  |  |  |  |  |  |  |  |  |  |  |  |  |  |  |  |
| IPN10 | 0 | 0 | 0.007 | 0 | 0 | 0.043 | 0.009 | 0.015 | 0.291 |  |  |  |  |  |  |  |  |  |  |  |  |  |  |  |  |  |  |
| IPN11 | -0.001 | 0 | 0 | 0 | 0 | 0 | 0 | 0.007 | 0.009 | 0.348 |  |  |  |  |  |  |  |  |  |  |  |  |  |  |  |  |  |
| IPN12 | 0 | 0 | 0 | 0 | 0 | 0 | 0 | 0 | 0 | 0.206 | 0.429 |  |  |  |  |  |  |  |  |  |  |  |  |  |  |  |  |
| IPN13 | -0.021 | 0 | -0.013 | -0.001 | -0.012 | 0 | 0 | 0.044 | 0.005 | 0.038 | 0.132 | 0.277 |  |  |  |  |  |  |  |  |  |  |  |  |  |  |  |
| IPN14 | -0.012 | -0.034 | 0 | 0 | 0 | 0 | 0 | 0.076 | 0.039 | 0.105 | 0.061 | 0.059 | 0.420 |  |  |  |  |  |  |  |  |  |  |  |  |  |  |
| IPN15 | 0.006 | 0 | 0.013 | 0.018 | 0.064 | 0 | 0.016 | -0.023 | 0.001 | 0 | 0.018 | 0.016 | 0 | 0.014 |  |  |  |  |  |  |  |  |  |  |  |  |  |
| IPN16 | 0 | 0 | 0 | 0 | 0 | 0 | 0.016 | -0.005 | 0 | 0 | 0 | 0 | -0.022 | 0 | 0.332 |  |  |  |  |  |  |  |  |  |  |  |  |
| IPN17 | 0.010 | 0 | 0.025 | 0.045 | 0.026 | 0 | 0.009 | 0 | 0 | 0 | 0 | 0 | -0.004 | 0 | 0.165 | 0.585 |  |  |  |  |  |  |  |  |  |  |  |
| IPN18 | -0.019 | 0 | 0.015 | 0.011 | 0.047 | 0 | 0.001 | 0 | 0.050 | 0.027 | 0.009 | 0 | -0.006 | 0.005 | 0.065 | 0.198 | 0.181 |  |  |  |  |  |  |  |  |  |  |
| IWB1 | -0.030 | -0.009 | 0 | 0 | -0.034 | 0 | 0 | 0 | 0 | 0 | 0 | 0 | 0.001 | 0 | -0.002 | 0 | 0 | 0 |  |  |  |  |  |  |  |  |  |
| IWB2 | 0 | 0 | 0 | -0.001 | -0.024 | 0 | 0 | 0 | 0 | 0 | 0 | 0 | 0 | 0 | 0 | 0 | 0 | -0.017 | 0.305 |  |  |  |  |  |  |  |  |
| IWB3 | 0 | -0.056 | 0 | -0.013 | 0 | 0 | 0 | 0 | 0 | 0 | 0 | 0 | 0.002 | 0.004 | 0 | 0 | 0 | 0 | 0.247 | 0.123 |  |  |  |  |  |  |  |
| IWB4 | 0 | 0 | 0 | -0.024 | -0.001 | -0.001 | 0 | 0 | 0.007 | -0.013 | 0 | -0.008 | 0 | 0 | -0.046 | 0 | 0 | 0 | 0.028 | 0.132 | 0 |  |  |  |  |  |  |
| IWB5 | -0.042 | -0.061 | 0 | -0.011 | -0.018 | 0 | 0 | 0 | 0 | 0 | 0 | 0 | 0 | 0.002 | 0 | 0 | -0.008 | 0.015 | 0.072 | 0.031 | 0.122 | 0.194 |  |  |  |  |  |
| IWB6 | -0.041 | -0.003 | -0.021 | 0 | -0.001 | 0 | 0 | 0 | 0 | -0.001 | 0 | 0.005 | 0.019 | 0 | 0 | 0 | -0.012 | 0 | 0.054 | 0.053 | 0.197 | 0.012 | 0.197 |  |  |  |  |
| IWB7 | 0 | 0 | 0 | -0.003 | -0.014 | 0 | -0.007 | 0 | 0 | 0 | 0.006 | 0 | 0 | 0 | 0 | -0.001 | -0.007 | 0 | 0.152 | 0.049 | 0.120 | 0.079 | 0.184 | 0.339 |  |  |  |
| IWB8 | -0.003 | -0.033 | 0 | -0.030 | -0.004 | -0.008 | -0.002 | 0 | 0 | 0 | 0 | 0 | 0 | 0.013 | 0 | -0.020 | -0.029 | -0.051 | 0.019 | 0.042 | 0.023 | 0.108 | 0.027 | 0.133 | 0.147 |  |  |
| IWB9 | -0.033 | 0 | -0.014 | 0 | -0.018 | 0 | 0 | 0 | 0 | 0 | 0 | 0 | 0.019 | 0 | 0 | 0 | -0.019 | 0 | 0.073 | 0.043 | 0.048 | 0.045 | 0.030 | 0.089 | 0.069 | 0.242 |  |

Table S2 Weighted edges among the 27 nodes of the lower-grade (a) and higher-grade (b) sample.

| (a) | | | | | | | | | | | | | | | | | | | | | | | | | | | |
| --- | --- | --- | --- | --- | --- | --- | --- | --- | --- | --- | --- | --- | --- | --- | --- | --- | --- | --- | --- | --- | --- | --- | --- | --- | --- | --- | --- |
|  | IPN1 | IPN2 | IPN3 | IPN4 | IPN5 | IPN6 | IPN7 | IPN8 | IPN9 | IPN10 | IPN11 | IPN12 | IPN13 | IPN14 | IPN15 | IPN16 | IPN17 | IPN18 | IWB1 | IWB2 | IWB3 | IWB4 | IWB5 | IWB6 | IWB7 | IWB8 | IWB9 |
| IPN1 |  |  |  |  |  |  |  |  |  |  |  |  |  |  |  |  |  |  |  |  |  |  |  |  |  |  |  |
| IPN2 | 0.671 |  |  |  |  |  |  |  |  |  |  |  |  |  |  |  |  |  |  |  |  |  |  |  |  |  |  |
| IPN3 | 0.015 | 0.132 |  |  |  |  |  |  |  |  |  |  |  |  |  |  |  |  |  |  |  |  |  |  |  |  |  |
| IPN4 | 0.050 | 0 | 0.525 |  |  |  |  |  |  |  |  |  |  |  |  |  |  |  |  |  |  |  |  |  |  |  |  |
| IPN5 | 0.055 | 0 | 0.169 | 0.300 |  |  |  |  |  |  |  |  |  |  |  |  |  |  |  |  |  |  |  |  |  |  |  |
| IPN6 | 0 | 0.004 | 0 | 0.002 | 0.108 |  |  |  |  |  |  |  |  |  |  |  |  |  |  |  |  |  |  |  |  |  |  |
| IPN7 | 0 | 0 | 0.010 | 0.014 | 0.017 | 0.436 |  |  |  |  |  |  |  |  |  |  |  |  |  |  |  |  |  |  |  |  |  |
| IPN8 | -0.016 | 0 | -0.013 | 0 | 0 | 0.235 | 0.323 |  |  |  |  |  |  |  |  |  |  |  |  |  |  |  |  |  |  |  |  |
| IPN9 | -0.005 | 0 | 0 | 0 | 0 | 0.046 | 0.144 | 0.288 |  |  |  |  |  |  |  |  |  |  |  |  |  |  |  |  |  |  |  |
| IPN10 | 0 | 0 | 0.013 | 0 | 0 | 0.021 | 0.009 | 0.035 | 0.283 |  |  |  |  |  |  |  |  |  |  |  |  |  |  |  |  |  |  |
| IPN11 | 0 | 0 | 0 | 0 | 0 | 0 | 0 | 0.015 | 0.013 | 0.335 |  |  |  |  |  |  |  |  |  |  |  |  |  |  |  |  |  |
| IPN12 | 0 | 0 | 0 | 0 | 0 | 0 | 0 | 0.003 | 0.026 | 0.205 | 0.449 |  |  |  |  |  |  |  |  |  |  |  |  |  |  |  |  |
| IPN13 | -0.041 | 0 | -0.015 | 0 | 0 | 0 | 0 | 0.024 | 0 | 0.072 | 0.124 | 0.252 |  |  |  |  |  |  |  |  |  |  |  |  |  |  |  |
| IPN14 | 0 | -0.023 | 0 | 0 | -0.003 | 0.021 | 0 | 0.065 | 0.032 | 0.071 | 0.069 | 0.049 | 0.465 |  |  |  |  |  |  |  |  |  |  |  |  |  |  |
| IPN15 | 0.004 | 0 | 0.005 | 0.008 | 0.063 | 0 | 0.007 | -0.012 | 0 | 0.012 | 0.019 | 0 | 0 | 0.007 |  |  |  |  |  |  |  |  |  |  |  |  |  |
| IPN16 | 0 | 0 | 0 | 0 | 0.001 | 0 | 0 | 0 | 0 | 0 | 0 | 0 | -0.005 | 0 | 0.398 |  |  |  |  |  |  |  |  |  |  |  |  |
| IPN17 | 0.012 | 0 | 0.037 | 0.055 | 0.031 | 0 | 0.018 | -0.010 | 0 | 0 | 0 | 0 | -0.015 | 0 | 0.184 | 0.516 |  |  |  |  |  |  |  |  |  |  |  |
| IPN18 | -0.025 | 0 | 0 | 0.016 | 0.049 | 0.010 | 0 | 0 | 0.041 | 0 | 0.015 | 0 | 0 | 0.002 | 0.052 | 0.214 | 0.203 |  |  |  |  |  |  |  |  |  |  |
| IWB1 | -0.038 | -0.017 | 0 | 0 | -0.021 | 0 | 0 | 0 | 0 | 0 | 0 | 0 | 0 | 0 | -0.003 | 0 | 0 | 0 |  |  |  |  |  |  |  |  |  |
| IWB2 | 0 | 0 | -0.012 | -0.005 | -0.003 | -0.003 | 0 | 0 | 0 | 0 | 0 | 0 | 0 | 0 | 0 | 0 | 0 | -0.003 | 0.278 |  |  |  |  |  |  |  |  |
| IWB3 | 0 | -0.047 | 0 | -0.009 | 0 | 0 | 0 | 0 | 0 | 0 | 0 | 0 | 0 | 0.004 | 0 | 0 | -0.002 | 0 | 0.272 | 0.122 |  |  |  |  |  |  |  |
| IWB4 | -0.007 | 0.011 | 0 | -0.012 | -0.017 | 0 | 0 | 0 | 0 | -0.011 | 0 | 0 | 0 | 0 | -0.036 | 0 | 0 | 0 | 0.032 | 0.135 | 0 |  |  |  |  |  |  |
| IWB5 | -0.042 | -0.036 | -0.005 | -0.005 | -0.017 | 0 | -0.001 | 0 | 0 | 0 | 0 | 0 | 0 | 0 | 0 | 0 | -0.005 | 0 | 0.043 | 0.028 | 0.129 | 0.246 |  |  |  |  |  |
| IWB6 | -0.070 | 0 | -0.018 | 0 | 0 | 0 | 0 | 0.004 | 0 | 0 | 0.003 | 0 | 0.011 | 0 | -0.012 | 0 | -0.006 | 0 | 0.054 | 0.051 | 0.223 | 0.013 | 0.202 |  |  |  |  |
| IWB7 | 0 | 0 | 0 | -0.032 | -0.006 | 0 | -0.005 | 0 | 0 | 0 | 0 | 0 | 0.002 | 0 | 0 | 0 | 0 | 0 | 0.151 | 0.058 | 0.122 | 0.091 | 0.174 | 0.309 |  |  |  |
| IWB8 | -0.011 | -0.037 | -0.001 | -0.011 | -0.015 | -0.001 | 0 | 0 | 0 | 0 | 0 | 0 | 0.007 | 0.011 | 0 | -0.020 | -0.028 | -0.039 | 0.016 | 0.071 | 0.010 | 0.095 | 0.055 | 0.126 | 0.135 |  |  |
| IWB9 | -0.013 | -0.001 | 0 | 0 | -0.047 | 0 | 0 | 0 | 0 | 0 | 0 | 0 | 0.018 | 0 | 0 | 0 | -0.003 | -0.017 | 0.085 | 0.016 | 0.040 | 0.033 | 0 | 0.088 | 0.116 | 0.242 |  |
| (b) | | | | | | | | | | | | | | | | | | | | | | | | | | | |
| IPN1 |  |  |  |  |  |  |  |  |  |  |  |  |  |  |  |  |  |  |  |  |  |  |  |  |  |  |  |
| IPN2 | 0.696 |  |  |  |  |  |  |  |  |  |  |  |  |  |  |  |  |  |  |  |  |  |  |  |  |  |  |
| IPN3 | 0.028 | 0.074 |  |  |  |  |  |  |  |  |  |  |  |  |  |  |  |  |  |  |  |  |  |  |  |  |  |
| IPN4 | 0.019 | 0 | 0.583 |  |  |  |  |  |  |  |  |  |  |  |  |  |  |  |  |  |  |  |  |  |  |  |  |
| IPN5 | 0.022 | 0 | 0.183 | 0.281 |  |  |  |  |  |  |  |  |  |  |  |  |  |  |  |  |  |  |  |  |  |  |  |
| IPN6 | 0 | 0 | 0 | 0 | 0.045 |  |  |  |  |  |  |  |  |  |  |  |  |  |  |  |  |  |  |  |  |  |  |
| IPN7 | 0 | 0 | 0 | 0 | 0.064 | 0.408 |  |  |  |  |  |  |  |  |  |  |  |  |  |  |  |  |  |  |  |  |  |
| IPN8 | -0.009 | -0.003 | 0 | -0.007 | 0 | 0.267 | 0.353 |  |  |  |  |  |  |  |  |  |  |  |  |  |  |  |  |  |  |  |  |
| IPN9 | -0.001 | 0 | 0.002 | 0 | 0 | 0.028 | 0.166 | 0.215 |  |  |  |  |  |  |  |  |  |  |  |  |  |  |  |  |  |  |  |
| IPN10 | 0 | 0 | 0 | 0 | 0 | 0.057 | 0.007 | 0.009 | 0.228 |  |  |  |  |  |  |  |  |  |  |  |  |  |  |  |  |  |  |
| IPN11 | -0.012 | 0 | 0 | 0 | 0 | 0.002 | 0 | 0.006 | 0.047 | 0.310 |  |  |  |  |  |  |  |  |  |  |  |  |  |  |  |  |  |
| IPN12 | -0.007 | 0 | 0 | 0 | 0.012 | 0 | 0 | 0 | 0 | 0.211 | 0.468 |  |  |  |  |  |  |  |  |  |  |  |  |  |  |  |  |
| IPN13 | -0.004 | -0.040 | 0 | 0 | 0 | 0 | 0 | 0.074 | 0 | 0.086 | 0.089 | 0.276 |  |  |  |  |  |  |  |  |  |  |  |  |  |  |  |
| IPN14 | -0.032 | -0.047 | 0 | 0 | 0 | 0 | 0.022 | 0.052 | 0.070 | 0.092 | 0.050 | 0.093 | 0.329 |  |  |  |  |  |  |  |  |  |  |  |  |  |  |
| IPN15 | 0 | 0 | 0.025 | 0.020 | 0.073 | 0 | 0.018 | -0.013 | 0.017 | 0.001 | 0 | 0.024 | 0 | 0.010 |  |  |  |  |  |  |  |  |  |  |  |  |  |
| IPN16 | 0 | 0 | 0 | 0.016 | 0.012 | 0 | 0 | -0.009 | 0 | 0 | 0 | 0 | -0.024 | 0 | 0.242 |  |  |  |  |  |  |  |  |  |  |  |  |
| IPN17 | 0 | 0 | 0.008 | 0.028 | 0.039 | 0 | 0.006 | 0 | 0 | 0 | 0 | 0 | 0 | 0 | 0.204 | 0.595 |  |  |  |  |  |  |  |  |  |  |  |
| IPN18 | -0.009 | 0 | 0.022 | 0.033 | 0.009 | 0.006 | 0.016 | 0 | 0.060 | 0.038 | 0.006 | 0 | 0 | 0.011 | 0.038 | 0.221 | 0.181 |  |  |  |  |  |  |  |  |  |  |
| IWB1 | -0.038 | -0.027 | 0 | -0.009 | -0.026 | 0 | -0.003 | 0 | 0 | 0 | 0 | 0 | 0 | 0 | 0 | 0 | 0 | 0 |  |  |  |  |  |  |  |  |  |
| IWB2 | 0 | -0.004 | 0 | 0 | -0.069 | 0 | 0 | 0.005 | 0 | 0 | 0 | 0 | 0 | 0 | 0 | 0 | 0 | 0 | 0.281 |  |  |  |  |  |  |  |  |
| IWB3 | -0.004 | -0.065 | 0 | 0 | 0 | 0 | 0 | 0 | 0 | 0 | 0.003 | 0 | 0.039 | 0.007 | 0 | 0 | 0 | 0 | 0.281 | 0.085 |  |  |  |  |  |  |  |
| IWB4 | 0 | 0 | 0.002 | -0.022 | -0.003 | 0 | 0 | 0 | 0.013 | 0 | 0 | 0 | 0 | 0 | -0.067 | -0.003 | 0 | 0 | 0.046 | 0.099 | 0 |  |  |  |  |  |  |
| IWB5 | -0.060 | -0.009 | 0 | -0.012 | -0.037 | -0.004 | 0 | 0 | 0 | 0 | 0 | 0 | 0 | 0 | 0 | 0 | -0.006 | 0 | 0.074 | 0.106 | 0.109 | 0.200 |  |  |  |  |  |
| IWB6 | -0.002 | -0.027 | -0.002 | -0.013 | -0.004 | 0 | 0 | 0 | 0 | 0 | 0 | 0.002 | 0.012 | 0 | 0 | -0.002 | -0.026 | 0 | 0.074 | 0.034 | 0.140 | 0.029 | 0.145 |  |  |  |  |
| IWB7 | -0.011 | -0.020 | 0 | 0 | 0 | 0 | -0.001 | 0 | 0 | 0 | 0.004 | 0 | 0 | 0 | -0.005 | 0 | -0.010 | 0 | 0.152 | 0.034 | 0.131 | 0.047 | 0.141 | 0.399 |  |  |  |
| IWB8 | 0 | -0.014 | -0.009 | -0.016 | -0.028 | -0.010 | -0.005 | 0 | 0 | 0 | 0.007 | 0.003 | 0 | 0 | 0 | -0.037 | -0.024 | -0.037 | 0 | 0.063 | 0.047 | 0.114 | 0.041 | 0.084 | 0.136 |  |  |
| IWB9 | -0.049 | -0.015 | -0.004 | 0 | 0 | 0 | 0 | 0 | 0 | 0 | 0 | 0 | 0 | 0 | -0.011 | 0 | -0.008 | 0 | 0.070 | 0.021 | 0.070 | 0.036 | 0.046 | 0.123 | 0.071 | 0.245 |  |

Table 3. Weighted edges among the 27 nodes of the whole sample.

|  | IPN1 | IPN2 | IPN3 | IPN4 | IPN5 | IPN6 | IPN7 | IPN8 | IPN9 | IPN10 | IPN11 | IPN12 | IPN13 | IPN14 | IPN15 | IPN16 | IPN17 | IPN18 | IWB1 | IWB2 | IWB3 | IWB4 | IWB5 | IWB6 | IWB7 | IWB8 | IWB9 |
| --- | --- | --- | --- | --- | --- | --- | --- | --- | --- | --- | --- | --- | --- | --- | --- | --- | --- | --- | --- | --- | --- | --- | --- | --- | --- | --- | --- |
| IPN1 |  |  |  |  |  |  |  |  |  |  |  |  |  |  |  |  |  |  |  |  |  |  |  |  |  |  |  |
| IPN2 | 0.695 |  |  |  |  |  |  |  |  |  |  |  |  |  |  |  |  |  |  |  |  |  |  |  |  |  |  |
| IPN3 | 0.012 | 0.11 |  |  |  |  |  |  |  |  |  |  |  |  |  |  |  |  |  |  |  |  |  |  |  |  |  |
| IPN4 | 0.046 | 0 | 0.553 |  |  |  |  |  |  |  |  |  |  |  |  |  |  |  |  |  |  |  |  |  |  |  |  |
| IPN5 | 0.048 | 0 | 0.172 | 0.3 |  |  |  |  |  |  |  |  |  |  |  |  |  |  |  |  |  |  |  |  |  |  |  |
| IPN6 | 0 | 0 | 0 | 0 | 0.087 |  |  |  |  |  |  |  |  |  |  |  |  |  |  |  |  |  |  |  |  |  |  |
| IPN7 | 0 | 0 | 0.012 | 0.003 | 0.034 | 0.428 |  |  |  |  |  |  |  |  |  |  |  |  |  |  |  |  |  |  |  |  |  |
| IPN8 | -0.018 | -0.001 | -0.013 | -0.001 | 0 | 0.249 | 0.339 |  |  |  |  |  |  |  |  |  |  |  |  |  |  |  |  |  |  |  |  |
| IPN9 | -0.004 | 0 | 0 | 0 | 0 | 0.036 | 0.151 | 0.263 |  |  |  |  |  |  |  |  |  |  |  |  |  |  |  |  |  |  |  |
| IPN10 | 0 | 0 | 0.006 | 0 | 0 | 0.038 | 0.006 | 0.026 | 0.267 |  |  |  |  |  |  |  |  |  |  |  |  |  |  |  |  |  |  |
| IPN11 | -0.002 | 0 | 0 | 0 | 0 | 0 | 0 | 0.012 | 0.032 | 0.323 |  |  |  |  |  |  |  |  |  |  |  |  |  |  |  |  |  |
| IPN12 | 0 | -0.004 | 0 | 0 | 0 | 0 | 0 | 0 | 0 | 0.215 | 0.461 |  |  |  |  |  |  |  |  |  |  |  |  |  |  |  |  |
| IPN13 | -0.042 | 0 | -0.003 | 0 | 0 | 0 | 0 | 0.045 | 0 | 0.074 | 0.111 | 0.263 |  |  |  |  |  |  |  |  |  |  |  |  |  |  |  |
| IPN14 | -0.003 | -0.039 | 0 | 0 | 0 | 0.007 | 0.01 | 0.059 | 0.049 | 0.077 | 0.061 | 0.065 | 0.419 |  |  |  |  |  |  |  |  |  |  |  |  |  |  |
| IPN15 | 0 | 0 | 0.014 | 0.015 | 0.069 | 0 | 0.017 | -0.027 | 0.01 | 0.01 | 0.012 | 0.009 | 0 | 0.013 |  |  |  |  |  |  |  |  |  |  |  |  |  |
| IPN16 | 0 | 0 | 0.001 | 0 | 0.008 | 0 | 0 | -0.007 | 0 | 0 | 0 | 0 | -0.019 | 0 | 0.339 |  |  |  |  |  |  |  |  |  |  |  |  |
| IPN17 | 0.005 | 0 | 0.026 | 0.044 | 0.029 | 0 | 0.014 | 0 | 0 | 0 | 0 | 0 | -0.009 | 0 | 0.186 | 0.556 |  |  |  |  |  |  |  |  |  |  |  |
| IPN18 | -0.025 | 0 | 0.01 | 0.025 | 0.035 | 0.012 | 0.003 | 0 | 0.049 | 0.008 | 0.015 | 0 | 0 | 0.008 | 0.045 | 0.219 | 0.193 |  |  |  |  |  |  |  |  |  |  |
| IWB1 | -0.035 | -0.02 | 0 | 0 | -0.023 | -0.001 | 0 | 0 | 0 | 0 | 0 | 0 | 0 | 0 | -0.003 | 0 | 0 | 0 |  |  |  |  |  |  |  |  |  |
| IWB2 | 0 | 0 | -0.001 | -0.004 | -0.028 | 0 | 0 | 0 | 0 | 0 | 0 | 0 | 0 | 0 | 0 | 0 | 0 | -0.007 | 0.285 |  |  |  |  |  |  |  |  |
| IWB3 | 0 | -0.059 | -0.001 | -0.004 | 0 | 0 | 0 | 0 | 0 | 0 | 0 | 0 | 0.012 | 0.006 | 0 | 0.004 | 0 | 0 | 0.278 | 0.106 |  |  |  |  |  |  |  |
| IWB4 | 0 | 0.01 | 0.019 | -0.025 | -0.012 | 0 | 0 | 0 | 0.007 | -0.006 | 0 | -0.009 | 0 | 0.004 | -0.051 | 0 | 0 | 0 | 0.039 | 0.124 | 0 |  |  |  |  |  |  |
| IWB5 | -0.052 | -0.031 | -0.005 | -0.01 | -0.027 | 0 | 0 | 0 | 0 | 0 | 0 | 0 | 0 | 0 | 0 | 0 | -0.008 | 0.009 | 0.051 | 0.057 | 0.125 | 0.228 |  |  |  |  |  |
| IWB6 | -0.044 | -0.003 | -0.018 | 0 | 0 | 0 | 0 | 0 | 0 | -0.003 | 0.002 | 0.004 | 0.014 | 0 | -0.007 | 0 | -0.019 | 0 | 0.062 | 0.044 | 0.192 | 0.022 | 0.177 |  |  |  |  |
| IWB7 | -0.006 | 0 | 0 | -0.019 | -0.001 | 0 | -0.008 | 0 | 0 | 0 | 0.003 | 0 | 0 | 0 | 0 | -0.002 | -0.001 | 0 | 0.154 | 0.047 | 0.126 | 0.074 | 0.162 | 0.352 |  |  |  |
| IWB8 | 0 | -0.028 | -0.013 | -0.006 | -0.015 | -0.01 | -0.002 | 0 | 0 | 0 | 0.003 | 0 | 0 | 0.01 | 0 | -0.026 | -0.025 | -0.047 | 0.008 | 0.069 | 0.026 | 0.108 | 0.048 | 0.111 | 0.135 |  |  |
| IWB9 | -0.03 | -0.008 | -0.003 | 0 | -0.027 | 0 | 0 | 0 | 0 | 0 | 0 | 0 | 0.015 | 0 | 0 | 0 | -0.011 | -0.002 | 0.081 | 0.016 | 0.051 | 0.034 | 0.012 | 0.097 | 0.101 | 0.244 |  |
